# Effect of Ghrelin on Glucose-Insulin Homeostasis: Therapeutic Implications

**DOI:** 10.1155/2010/234709

**Published:** 2010-02-09

**Authors:** Susana Sangiao-Alvarellos, Fernando Cordido

**Affiliations:** ^1^Department of Medicine, School of Health Science, University of A Coruña, Xubias de Arriba 84, 15006 A Coruña, Spain; ^2^Institute of Biomedical Investigations (INIBIC), Group of Endocrinology, Xubias de Arriba, 84, 15006 A Coruña, Spain; ^3^Department of Endocrinology, Complexo Hospitalario Universitario A Coruña, Xubias de Arriba, 84, 15006 A Coruña, Spain

## Abstract

Ghrelin is a 28-amino-acid peptide that displays a strong growth hormone- (GH-) releasing activity through the activation of the growth hormone secretagogue receptor (GHSR). The first studies about role of ghrelin were focused on its orexigenic ability, but despite indisputable pharmacological data, the evidence for a physiological role for ghrelin in the control of appetite is much less clear. Mice with targeted deletion of either ghrelin or the GHSR exhibit an essentially normal metabolic phenotype when fed a regular chow diet, suggesting that ghrelin may have a redundant role in the regulation of food intake. RNAs for ghrelin as well as GHSR are expressed in the pancreas of rats and humans and several studies propose that ghrelin could have an important function in glucose homeostasis and insulin release, independent of GH secretion. Low plasma ghrelin levels are associated with elevated fasting insulin levels and insulin resistance, suggesting both physiological and pathophysiological roles for ghrelin. For this reason, at least theoretically, ghrelin and/or its signalling manipulation could be useful for the treatment or prevention of diseases of glucose homeostasis such as type 2 diabetes.

## 1. Introduction

GH is released from the pituitary gland in a pulsatile manner and it is mainly regulated by episodic changes in two hypothalamic hormones, growth hormone-releasing hormone (GHRH) and somatostatin. GHRH stimulates GH secretion whereas that somatostatin inhibits it [1]. In 1976, it was revealed that modified opioid peptides had low GH secretory activity [2]. Since then, many efforts have been made to develop and improve potential applications of these GH secretagogues (GHSs) [3–7]. GHSs act on the pituitary and hypothalamus to release GH, not through the growth hormone releasing hormone receptor (GHRHR) but through an orphan receptor, the GHSR [8]. These facts indicated that an unknown endogenous ligand for GHSR should exist. In 1999, ghrelin was identified as the endogenous ligand for the GHSR. It is a 28-amino-acid peptide predominantly produced by the stomach that functions as a somatotrophic and orexigenic signal from the stomach [9, 10]. Ghrelin is most abundantly expressed in specialized cells in the oxyntic glands of the gastric epithelium, originally termed X/A-like cells [11]. Approximately 60%–70% of circulating ghrelin is secreted by the stomach, and most of the remainder originates in the small intestine [11]. Nevertheless low-level ghrelin expression also occurs in several tissues outside the gut, including hypothalamus (arcuate nucleus and paraventricular nucleus), pituitary, lung, adrenal cortex, kidney, bone, testis, placenta, and pancreatic islet cells [12]. The GHSR mRNA is expressed as two splice variants encoding the cognate receptor GHSR1a and the apparently nonfunctional receptor GHSR1b [13]. GHSR1a signals via inositol trisphosphate (IP_3_) generation and Ca^2+^ release and has constitutive activity [13, 14]. GHSR1b mRNA is as widely expressed as ghrelin, whereas GHSR1a gene expression is concentrated in the hypothalamus-pituitary unit, although it is also distributed in other central and peripheral tissues [15]. Ghrelin circulates in the bloodstream in two different forms: acylated (or n-octanoylated, AG) and unacylated (or des-octanoylated or des-acylated, UAG) [9]. AG has a unique feature: a posttranslational esterification of a fatty (n-octanoic or, to a lesser extent, n-decanoic) acid on serine residue at position 3 [9]. Recent data showed that Ghrelin O-acyltransferase (GOAT), a membrane-bound enzyme, is responsible for octanoylation of the serine-3 residue of ghrelin [16, 17]. Ghrelin acylation is considered necessary for its actions via GHSR1a, such as its strong GH-releasing activity [9, 18–20]. Normally AG accounts for less than 10% of the total ghrelin in the circulation. The majority of circulating ghrelin is UAG, which does not have effects in GH release, but it is not biologically inactive [19, 21–29]. It binds with high affinity to a receptor, different from GHSR1a and yet unknown [9, 12]. The first studies about ghrelin demonstrated that it increases food intake and adiposity [10]. Moreover, plasma ghrelin levels have been shown to increase prior a meal and during fasting and to decrease after a meal, and they are negatively correlated with body weight [30–32]. All these data suggested a role in the control of energy homeostasis. But the conflicting food intake and body weight data from transgenic and knockout models, which present normal metabolic phenotype, has made difficult defining a key role for endogenous ghrelin in the control of appetite [27, 33–39]. Nevertheless, the data consistently suggest that ghrelin may be important in the control of glucose homeostasis and insulin release. 

 It was reported that prolonged treatment with GHSs provoked hyperglycemia and hyperinsulinism but this effect was supposed to reflect increased GH secretion [40–43], as GH plays an important role modulating energy homeostasis and metabolism [44]. Particularly, GH exerts both acute and chronic effects on carbohydrate and lipid metabolism [44]. Interestingly, both actions display an opposite pattern, with acute effects showing a transient “insulin-like” action and chronic effects exhibiting an “anti-insulin” action. In this sense, GH administration decreases blood glucose concentration, stimulates glucose uptake by skeletal muscle, and stimulates glucose transport and lipogenesis in isolated adipocyte [44, 45]. However these effects are transitory; after a few hours the chronic anti-insulin effects of GH arise, increase blood glucose concentration, insulin resistance, stimulation of lypolysis, and inhibition of glucose transport. High plasma GH levels induce hyperinsulinemia and insulin resistance [12, 46]. On the other hand, besides its lipolytic action, GH exhibit antilipogenic effects [47, 48] promoting proliferation of lean tissues, while reducing accumulation of fat tissue. Accordingly, GH-deficient states in humans and rodents are characterized by a decrease in lean body mass accompanied by increased adipose tissue [49–53]. 

Early studies demonstrated that RNAs for ghrelin, as well as GHSR, are expressed in the pancreas of rats and humans [9, 15, 54, 55] and *β*-cells lines [56, 57] suggesting a possible relation between ghrelin and insulin. Studies using different experimental systems localized ghrelin-immunoreactive cells in rat and human pancreas in the *α*-cells [54, 57, 58], *β*-cells [55], PP-cells [57], and other islets cells [57, 59], including those named *ε*-cells [60]. The first evidences about an interaction between ghrelin and glucose metabolism arose when it was seen that single subcutaneous ghrelin injections induced an increase of the respiratory quotient (RQ), which suggested an augmented utilization of carbohydrate and reduced utilization of fat to meet energy requirements that was congruent with the observed increase in body fat [10]. Another evidence that suggested that ghrelin could affect glucose metabolism was the fact that it stimulated acid secretion through vagal mediation [61, 62] and some studies suggested that the parasympathetic nerves that regulate hormonal control of insulin pass through the cervical vagus and the hepatic branch, and that the hepatic vagus nerve is important for the regulation of hepatic glucose production in the post absorptive state [63, 64]. All these data and numerous studies since 2000 to the present time suggest that ghrelin has an important role in regulating *β*-cell function and glucose homeostasis. Indeed, the weight of evidences could support even a more physiologically important function in the control of glucose homeostasis than appetite regulation. 

 In this work we will review the results obtained by different investigators about the relation between ghrelin and glucose metabolism and insulin release as well as its possible therapeutic role in disease states like diabetes.

## 2. Effects of Exogenous Ghrelin on Glucose and Insulin Levels

### 2.1. Short-Term Effects

The first studies with ghrelin showed that acute ghrelin treatment induced hyperglycemia and reduced insulin secretion in healthy humans [65] during the first hours of treatment. The time course of glucose modifications occurred with a peak observed before any significant insulin decreased. Subsequently, these findings were confirmed by other authors in human and rodents [58, 66–71]. However, when these experiments were carried out in obese patients, there was no difference in glucose or insulin levels following ghrelin administration [72]. The first hypothesis suggested that ghrelin itself could have a direct effect on glucose metabolism, regulating hepatic glucose output, promoting glycogen breakdown, or decreasing peripheral glucose uptake; consistent with this view were the findings that ghrelin receptors are expressed in normal human liver [73, 74]. 

 In order to discard a secondary effect due to increased GH secretion, human subjects were treated with a GH receptor blockade, pegvisomant [43], and in this situation a ghrelin mimetic induced increases in glucose and insulin levels. Suggesting ghrelin mimetic-mediate GH-independent insulin resistance, moreover several studies have demonstrated that interference with ghrelin signalling by use of GHSR antagonist decreases blood glucose in wild-type mice as well as GH-deficient lit/lit mice [58, 75]. To diminish the influence of GH, Vestergaard et al. investigate the effects of prolonged ghrelin infusion (not a unique dose) on insulin sensitivity [76] which decreased after few minutes of ghrelin infusion and outlasted both the infusion period and the postinfusion interval. As the reduced insulin sensitivity remained after normalization of both GH and glucose levels, this work supports that ghrelin effect was caused by the ghrelin infusion per se [77]. In a posterior work the same investigators studied, for the first time, the concomitant effects of exogenous ghrelin and a pancreatic clamp on glucose metabolism in humans; they used a prolonged ghrelin infusion in addition to a somatostatin infusion to avoid GH secretion. Ghrelin infusion decreased basal as well as insulin stimulated glucose disposal and induced peripheral insulin resistance but did not affect hepatic glucose production [71]. When they studied the effect of exogenous ghrelin in hypopituitary men (in the absence of GH and cortisol secretion), in a randomized double-blind, cross-over design, ghrelin treatment acutely decreased peripheral, but not hepatic, insulin sensitivity independently of GH and cortisol [78]. 

 There are data that suggest a relation between ghrelin and glucose-stimulated insulin secretion (GSIS) via the hepatic portal system and the vagus nerve. Gastrectomy and truncal vagotomy are operations characterized by hypoghrelinemia [30], glucose intolerance as a result of hyperglucagonemia, insulinopenia, and impaired first phase of insulin secretion [79]. When ghrelin was infused into the portal vein of rats, inhibited glucose-stimulated release of insulin, however when it was infused into the femoral vein, did not induce such an inhibitory effect. All the more hepatic vagotomy or coinfusion with atropine methyl bromide (a muscarinic antagonist) diminished the inhibitory effect of ghrelin on glucose-stimulated insulin secretion [70]. Damjanovic et al. also performed studies with ghrelin and truncal vagotomy, investigating the effects of intravenous (IV) ghrelin infusion on insulin-mediated glucose disposal during a hyperinsulinemic-euglycemic clamp in humans who underwent total gastrectomy and truncal vagotomy [80]. In these patients glucose disposal rate (GDR) decreased during ghrelin infusion; however this difference was not translated into a significant difference in insulin concentration, probably because the exogenous insulin by far overweighs endogenous insulin. Thus, there cannot be ruled the possibility that diminished glucose utilization after ghrelin administration is partly explained by the decrease in endogenous insulin secretion, although this was not detected in the study [80]. It appears that acute ghrelin administration might be involved in the negative control of insulin secretion and glucose consumption in gastrectomized patients [80].

 In summary, short-term effects of exogenous ghrelin induces hyperglycaemia and hypoinsulinism in health humans and rodents in a GH independent fashion. 

 In Table 1 are summarized the results obtained after acute ghrelin treatment in several models and situations.

### 2.2. Long-Term Effects

Generally long-term ghrelin treatment induced an increase in plasmatic values of glucose, whereas plasmatic insulin levels, unlike short-term effects, did not change or enhanced after ghrelin treatment. But long-term effects of exogenous ghrelin on glucose and insulin levels are not conclusive; there are differences inter-experiments which could reflect different doses, administration way, and/or species used. In long term studies essentially there are two way of administration: those in which the administration of ghrelin was systemic: intraperitoneal (IP) or subcutaneous (SC), and central: when the hormone was administered directly in a cerebral region.

#### 2.2.1. Systemic Administration

Involves treated IP with ghrelin during 4 days, plasma glucose concentrations increased. At the same time, the authors measured body glycogen stores and observed that liver glycogen content was unaffected, but the quadriceps muscle and kidney glycogen stores decreased, indicating them as the possible source of elevated plasma glucose levels [69]. Similar results were obtained by Asakawa et al. with mice; they examined the effects of repeated administration of IP ghrelin on glycaemic control under a high fat diet (HFD). In these conditions insulin levels were increased by the treatment and blood glucose concentration displayed a moderate increase but did not reach statistical significance [75]. 

 When Barazzoni et al. administrated subcutaneous ghrelin during four days to normal rats, they found hyperglycemia; nevertheless plasma insulin levels did not change [85, 86]. The treatment increased transcript levels of the key enzyme of the gluconeogenic pathway, glucose-6-phosphatase (G6Pase) in liver. For these reasons the authors suggested that enhanced gluconeogenesis in liver would contribute to increase circulating glucose in ghrelin-treated animals [85].

#### 2.2.2. Central Administration

In others studies the animals received ghrelin treatment intracerebroventricularly (ICV). 6-day ICV ghrelin infusion provoked an increase on insulin-stimulated glucose utilization during euglycemic-hyperinsulinemic clamps in epididymal and inguinal white adipose tissue (WAT) as well as brown adipose tissue (BAT), but not in soleus muscle. During the clamps, hepatic glucose production was comparably suppressed by hyperinsulinemia in all groups. The treatment did not change plasma glucose or insulin levels [87]. Comparable results were obtained by Kamegai et al. administering repeated injections of ghrelin into the lateral ventricle of rats during 72 hours, without changes in plasma glucose and insulin concentrations, although there was a trend toward higher levels [88]. However, in another study, ICV ghrelin injections every 24 hours during five days to adult male rats clearly increased serum insulin levels without evoking changes in blood glucose levels [89]. 

 Although the results obtained by ghrelin treatment in the long term are not enough clear, it seems to exist a tendency toward an increase in both plasma glucose and insulin levels. These data could indicate a role for ghrelin in worsening insulin sensitivity. 

 In Table 2 are summarized the results obtained in plasma glucose and insulin levels after prolonged treatment with ghrelin.

### 2.3. Studies In Vitro and Perfusion

Besides the experiments carry out in vivo, there are works with cellular cultures and pancreatic perfusion that contribute to our knowledge about ghrelin role on glucose and insulin metabolism, pointing to a role for ghrelin in the pancreatic islet. The perfused rat pancreas is a suitable model to characterize the pancreatic hormone secretory pattern elicited by ghrelin in the short term. Egido et al. dissected and perfused in situ the pancreas of rats fed *ad libitum*; the addition of ghrelin to the perfusate did not significantly modify basal insulin release but markedly inhibited the insulin response to increasing glucose concentrations, arginine, and carbachol [90]. It was observed that the glucose-induced insulin release from the rat-perfused pancreas was markedly enhanced by blockade of GHSR and immunoneutralization of endogenous ghrelin. Furthermore, GHSR blockade increased plasma insulin concentrations in gastrectomized and normal rats to a similar extent [91]. The results obtained with perfused rat pancreas support a role for ghrelin inhibiting insulin release. These results were confirmed in studies with isolated islets from normal rats [58, 92] and MIN 6 cells [93], where ghrelin inhibited the insulin response to increasing glucose concentrations. But when ghrelin was coincubated with GHSR antagonists or antiserum against acylated ghrelin, this effect was blocked [58, 92]. Moreover, in islets from ghrelin-null mice, glucose treatment enhanced insulin release [91]. On the contrary, in another study, it was observed that ghrelin (1 pmol/l) stimulated insulin release and increased [Ca^2+^] in rat islet *β*-cells in the presence of a stimulatory (8.3 mmol/l) but not basal (2.8 mmol/l) glucose concentration [54]. However, the same authors, in a subsequent study, examined the dose-dependent effects of ghrelin and they found that ghrelin at 1 pmol/l and 0.1 nmol/l modestly potentiated glucose-induced [Ca^2+^]i responses in a little portion of *β*-cells, but it failed to significantly alter insulin release. This observation that ghrelin is inhibitory at relatively high concentrations of 10 nmol/l, while having little effect at lower concentrations, is consistent with the majority of other reports [58]. 

 Several cell culture studies showed a genetic link between ghrelin and insulin. The Nkx2.2 hoursomeodomain transcription factor is required for islet cell development and differentiation. In this way high levels of Nkx2.2 are necessary to specify or maintain the islet *β* cell fate [94]. Nkx2.2 null mice completely lack insulin-producing *β*-cells and have reduced numbers of *α*-cells. In normal islets, a population of glucagon-expressing *α*-cells coexpress ghrelin, but approximately two-thirds of ghrelin-expressing cells define a new endocrine islet, *ε* cell population. In addition, in the Nkx2.2 mutant islet, the ghrelin-producing *ε* cell population has been drastically expanded at the expense of insulin- and glucagon-producing cells. [60]. Similar to the wild-type islet, ghrelin producing cells in the Nkx2.2 mutant embryonic mouse islets do not coexpress insulin, somatostatin, or PP. However, unlike its expression in wild-type islets, none of the ghrelin-producing cells in the Nkx2.2 mutant coexpress glucagon [60]. 

 On the other hand, insulinoma-associated protein (IA)-2*β* is a *β*-cell autoantigen for type 1 diabetes. It is localized in secretory granules in pancreatic *β*-cells or neuroendocrine cells [95]. Stable overexpression of IA-2*β* inhibited GSIS in MIN6 cells when performed in medium containing glucose. Doi et al. observed that ghrelin inhibits GSIS in MIN6 cells and that the concentrations of ghrelin inhibiting GSIS were very close to those of ghrelin enhancing IA-2*β* expression, suggesting that ghrelin may inhibit GSIS via enhancement of IA-2*β* expression [93]. Incubation of cultured MIN6 cells with increasing doses/times of ghrelin showed that ghrelin induced IA-2*β* RNA and protein expression dose dependently. The blockage of IA-2*β* expression with siRNA provoked that the inhibitory effects of ghrelin or overexpression of IA-2*β* on GSIS were ameliorated, providing direct evidence of the links between ghrelin, IA-2*β*, and GSIS; changes in insulin content in the cell lysates or in insulin mRNA expression were not observed [93]. 

 Some of the results obtained with this type of techniques are displayed in Table 3. 

### 2.4. Unacylated Ghrelin

Acylated ghrelin accounts for less than 10% of the total ghrelin; the majority of circulating ghrelin is unacylated. Although UAG does not possess GH releasing activity, it is not biologically inactive. Several studies demonstrated a clear metabolic role for UAG; it is able to share with ghrelin antiproliferative effects on human breast and prostate cancer lines [97, 98], has negative inotropic effects on papillary muscle [99], and can stimulate bone marrow adipogenesis [28]. These effects of UAG could not be antagonized by administration of synthetic GHSR1a antagonists [28] as UAG is unable to bind the classical GHSR1a, which recognizes ghrelin in its acylated form only [9]. The signal transduction mechanism(s) for effects of UAG has not been determined. Evidences that UAG is an active peptide implies the existence of GHSR subtypes that recognize and bind ghrelin independently of its acylation. These binding sites have already been demonstrated in the cardiovascular system and in the pancreas [21, 98, 100]. Besides the effects above mentioned, several studies suggested a role of UAG on glucose metabolism. Broglio and colleagues suggested that ghrelin could have a dualistic effect on glucose homeostasis; its effect on insulin secretion and sensitivity could depend on its state of acylation. They observed that in healthy humans, the administration of UAG alone did not induce any change in glucose and insulin levels compared to placebo. Nevertheless UAG counteracts the effects of AG on glucose and insulin levels, but not its stimulatory action on GH, PRL, ACTH, and cortisol levels, indicating that UAG has metabolic impact, being able to antagonize the effects of AG on insulin and glucose levels, while it is really inactive from the neuroendocrine point of view [67]. 

 Similar results were obtained in humans with pituitary insufficiency. In these patients both AG and UAG immediately increase glucose and insulin levels, when AG and UAG were injected together; this combination prevents the acute hyperglycaemic and hyperinsulinemic effects of AG and UAG when injected alone. Moreover, this combination of AG and UAG improves insulin sensitivity for many hours when compared with placebo administration and even more markedly with the aggravation of insulin sensitivity of AG administration [82]. 

 As both AG and UAG are secreted into the portal circulation before they reach the systemic circulation, and the above reported effects of AG and UAG on glucose and insulin levels in vivo are based on measurements of systemic blood samples. Gauna et al. hypothesized that, concerning insulin secretion, assessment of insulin concentration in the portal vein might be more informative than that in the systemic circulation. They demonstred in anesthetized rats that UAG acted as a secretagogue of insulin in the portal vein. Moreover, this UAG-induced increase in insulin levels was abolished by the coadministration of AG. This study showed that UAG potently and dose-dependently enhances the insulin response to an intravenous glucose load in vivo [83]. This insulin secretagogue effect of UAG was marked in the portal vein, whereas it was scarcely detectable in the systemic circulation, suggesting that UAG plays an important role in glucose metabolism in the liver. Gauna et al. estimated that UAG slightly increased the fraction of insulin cleared by the liver, thus contributing to the augmentation of the portal-peripheral gradient of insulin [83]. Furthermore several studies support the possibility that ghrelin has a direct peripheral action on liver [73, 96]. Recently ghrelin levels have been found decreased in liver failure patients [101], a clinical condition with altered nutrition and glucose homeostasis. When Gauna et al. studied the effects of AG and UAG on primary hepatocytes; they confirmed that ghrelin in vitro induces a rapid increase of glucose output by primary hepatocytes, which suggests that AG modulates glucose homeostasis at least by acting directly on the liver. It was found that UAG itself exerts an inhibitory effect on glucose output and; as was seen in normal subjects in vivo, it is able to counteract the inductive effect of AG on glucose release [96]. The results obtained by different authors appear to indicate that the administration of UAG in humans might improve insulin sensitivity and secretion in subjects with relative or absolute GH deficiency and in the presence of GH. 

 These effects of UAG in the regulation of glucose metabolism might be of therapeutic interest for those pathological conditions characterized by insulin resistance and impaired insulin release.

## 3. Effects of Endogenous Ghrelin on Glucose and Insulin Levels

### 3.1. Studies In Vivo with GHSR Antagonists

In order to study the effects of endogenous ghrelin on glucose and insulin metabolism, many investigators used GHSR antagonists like modified GHRP-6 or YIL-781. In normal mice blockade of endogenous ghrelin by intraperitoneal injection of modified GHRP-6 markedly lowered fasting glucose concentrations in a few hours. Similarly during the intraperitoneal glucose tolerance test (IP-GTT), plasma glucose elevation was attenuated and insulin response was enhanced, showing a physiological role for endogenous ghrelin in the regulation of insulin release and blood glucose [58]. On the other hand YIL-781 did not affect fasting blood glucose levels. But, upon IP-GTT, the compound as well as modified GHRP-6 caused a decrease in the glucose excursion relative to the vehicle-treated animals. During an insulin tolerance test (ITT), YIL-781 did not alter the effect of insulin on blood glucose levels. This result, in combination with the effect of the compound on insulin secretion, demonstrates that, at least acutely, the GHSR1a antagonist YIL-781 improves glucose tolerance by promoting insulin release rather than enhancing insulin sensitivity. To evaluate whether GHSR1a antagonists could improve glucose tolerance in a disease model, YIL-781 was tested in the insulin-resistant diet-induced obesity (DIO) rat. In this model an oral dose of YIL-781 causes a reduction in glucose excursion. [92]. The data obtained by Esler et al. provide evidence that GHSR1a antagonists had no apparent effect on insulin sensitivity but improved glucose tolerance by stimulating insulin secretion. When ob/ob obese mice, which are a known genetic model of obesity and diabetes with insulin resistance, were peripherally administered with modified GHRP-6 during several days, plasma glucose levels diminished. This reduction in glucose levels was accompanied by a moderate decrease in serum insulin levels, suggesting that GHSR antagonists ameliorated insulin resistance in the long term [75]. 

 The data obtained with GHSR1a antagonists (summarized in Table 4) suggest that these drugs could improve glucose tolerance and ameliorate insulin resistance in the long term and hence may be promising targets for pharmacological intervention in the treatment of type 2 diabetes.

### 3.2. Glucose and Insulin Levels in GHSR-, Ghrelin-, and Double-Knockout Animals

The knockout (KO) animals represent a good opportunity to study endogenous ghrelin functions. Plasma ghrelin concentration is inversely correlated with body weight and body fat [102]. Moreover, considering that one of the main characteristics of exogenous ghrelin is to increase food intake, body weight, and % body fat [10, 87] it was expectable that the null animal for ghrelin and/or GHSR had marked differences in the ingestion and/or body composition; however the results obtained did not show that. It seems that the type of diet, its duration, age, and nutritional status of the animals are key factors to understand the function of the hormone in the energetic metabolism as well as its effect in the homeostasis of glucose and insulin. The results obtained in rats in relation to the metabolism of glucose and insulin in knockout animals are shown in Table 5.

#### 3.2.1. GHSR Knockout

Some investigators reported that GHSR knockout animals, in comparison with wild-type controls, had only a modest decrease in body weight when maintained on standard chow and similar levels of insulin in both fed and fasted states [106]. However GHSR null mice to 50% caloric restriction (CR) or fasting conditions on standard diet had lower blood glucose and insulin levels than standard diet fed wild-type (WT) mice suggesting enhanced insulin sensitivity [34]. These results were supported by other authors. Zigman et al. also observed that GHSR-null male mice showed lower blood glucose levels when maintained on a standard chow diet (SCD), and corresponding insulin levels were lower, although not always reached statistical significance [104]. 

 It was observed that GHSR null mice had mean body weight and body composition comparable to those of their same-sex wildtype littermates when measured 1 week after weaning or exposure to standard chow. However, several weeks of exposure to HFD after weaning resulted in significantly less accumulation of both body weight and body fat content in GHSR null mice, as compared with littermate controls, and these animals presented resistance to diet-induced obesity [33, 104]. Interestingly, these differences are masked in HFD fed mice only in their adult stage; in this situation the deletion of GHSR does not prevent DIO or weight gain after weight loss [34]. 

 Once more, in GHSR null mice fed with HFD, several measures of greater insulin sensitivity were observed, including lower fasted blood glucose and plasma insulin, lower insulin levels during glucose tolerance tests, and improved performance in hyperinsulinemic-euglycemic and hyperglycemic clamp studies [33]. 

 On the other hand, the results obtained in RQ for GHSR null mice are discrepant. The knockout created by Nakano et al. presented decreased RQ during long-term HFD study that represents a shift in metabolic fuel preference toward the utilization of fat as an energy substrate [104]. On the contrary, Longo's animals have higher RQ, indicating a preference for carbohydrate as fuel regardless of gender or diet. These data could suggest that ghrelin's effects on metabolic fuel preference are transient and may not have a significant effect throughout the lifespan. Perhaps adult GHSR null mice are subject to metabolic adaptations especially in regard to energy intake and expenditure. However the range of RQ values was wider in knockout mice, indicating greater metabolic flexibility in these animals [33].

#### 3.2.2. Ghrelin Knockout

When ghrelin KO animals and WT controls were exposed to prolonged and earlier HFD (after weaning), ghrelin KO mice showed mean body weight and mean body fat percentage that were lower than those of similarly treated wild-type controls [107]. This diet produced glucose intolerance and insulin resistance in wild type mice [91, 107]. By contrast, ghrelin knockout mice fed with HFD showed close to normal glucose responses and markedly enhanced insulin responses to IP-GTTs compared with control ghrelin knockout mice fed with SCD [91]. As a possible underlying mechanism Dezaki et al. suggested that lack of ghrelin and its insulinostatic activity may raise the maximal capacity of glucose-induced insulin release and enable islets to secrete more insulin to meet an increased demand associated with HFD–induced obesity, thereby achieving normoglycemia [91]. Moreover Ghr KO mice on the HFD presented lower levels of glucose and insulin as well as lipids compared with wild-type on this diet; hence ghrelin as well as GHSR null mice exposed to HFD after weaning exhibit greater glucose tolerance. The results of GTTs and ITTs were similar to those previously observed for the same authors with pharmacological blockade of ghrelin action [58], reinforcing the concept that endogenous ghrelin serves as a regulator of insulin release and of glycemia. However, when ghrelin null mice and wild type mice were subjected to acute exposure to HFD late in life, slight differences in body composition between ghrelin KO animals and wild-type controls were reported, and no change in glucose and insulin levels [103]. Comparable results were obtained in animals fed with standard chow, where insulin and glucose levels did not display changes [35]. Moreover these animals did not display differences in cumulative food intake on standard chow or body weight change and food intake in response to reexposure to food following a fast [35]. However, Sun et al. realized several studies where they observed, that compared to WT, ghrelin KO mice exhibited significantly lower glucose levels after IP-GTT and correspondingly higher levels of insulin. In addition, the initial insulin response at 15 minutes was significantly higher in the ghrelin KO compared to WT mice [36]. When ghrelin KO mice were subjected to 50% caloric restriction, they had lower blood glucose levels than their WT littermates suggesting that ghrelin would be involved in providing a counterregulatory glucose response during negative energy balance [34]. 

 In another line of ghrelin knockout mice, glucose levels were monitored in lean mice (wild-type and ghrelin KO) and obese mice (wild type ob/ob and ghrelin KO ob/ob) at different ages [36]. The lean mice were euglycemic; as expected, glucose and insulin levels were elevated both in ob/ob and ghrelin KO ob/ob mice. However blood glucose was elevated at age 4 weeks in ob/ob mice and at 6 weeks in ghrelin KO ob/ob mice, and although obesity was as severe as in ob/ob mice, ghrelin KO ob/ob exhibited lower glucose levels and their blood glucose normalized upon fasting. Hence, ablation of ghrelin markedly improved glucose homeostasis in ob/ob mice [36]. The improvement in glucose homeostasis in ghrelin KO ob/ob mice was accompanied by increased serum insulin levels. Remarkably, compared to ob/ob mice, ghrelin KO ob/ob mice displayed reduced blood glucose concentrations after IP-GTT, which was accompanied by increased insulin secretion [36]. When ghrelin KO mice, maintained from weaning on regular chow, were subjected to IP-GTT, ghrelin treatment produced higher blood glucose and markedly lowers insulin levels, showing that ghrelin acutely suppresses insulin release, suggesting that the improved glucose tolerance which was observed in ghrelin KO ob/ob mice fed with HFD during IP-GTT could be a consequence of ghrelin-ablation. Moreover, ghrelin ablated mice presented greater reductions in glucose levels 30 minutes following ITT suggesting increased insulin sensitivity. When the authors subjected WT and ghrelin KO mice to euglycemic hyperinsulinemic clamp studies, basal hepatic glucose production rate was the same in both genotypes. But during the low-dose insulin clamp, suppression of glucose production was higher in ghrelin KO mice, proposing once more that the liver of ghrelin KO mice was more sensitive to insulin. Furthermore, an increase in glucose infusion rate (GIR) and an increase in GDR were detected, indicating that besides increasing glucose-induced insulin secretion, ghrelin ablation increased peripheral insulin sensitivity and improves glucose tolerance [36]. 

 Wortley et al. found a trend toward decreased weight and leaner body composition in male ghrelin knockout mice after 6 weeks on the HFD, which could be explained by a decrease in RQ observed only in these animals; therefore the constitutive absence of ghrelin causes a distinct shift toward lipid metabolism during consumption of an HFD [103].

#### 3.2.3. Ghrelin/Ghrelin Receptor Double Knockout (dKO) Mice

Pfluger and colleagues created ghrelin/ghrelin receptor double knockout mice. Plasma glucose and plasma insulin levels did not differ between aged WT and dKO mice after an overnight fast. An IP-GTT overall failed to reveal significant differences in glucose tolerance between genotypes. Mice deficient in either ghrelin, GHSR, or both showed lower glucose peak levels at a single time point (15 minutes after the injection) suggesting a slightly faster release of insulin. Mice were subjected to an ITT; in ghrelin KO mice glucose levels were similar to WT mice. In dKO and GHSR KO mice, glucose levels, however, dropped more rapidly. In general, glucose levels of dKO and GHSR mice tended to remain lower throughout the 120 minutes of the ITT, compared with WT mice. However, although integrated glucose levels in both GHSR KO and dKO mice tended to be lower compared with WT control mice, the deficiency of ghrelin, its receptor, or both did not seem to have a major impact on overall insulin sensitivity or the overall regulation of glucose homeostasis. They observed substantial but mostly insignificant trends in glucose tolerance and insulin sensitivity. Importantly, all these data were obtained from mice maintained on normal standard chow diet [37]. 

 Pfluger et al. speculated that their mouse mutants still may exhibit some level of ghrelin signaling, although by definition they genetically deleted ghrelin [9], its putative ghrelin associated peptide [108], ghrelin splice variants [109], and the constitutively active ghrelin receptor GHSR [110]. For this reason the authors suggested that the existence of both additional ligand and additional receptor, coded for by genes other than the ghrelin and the GHSR gene, could explain why the dKO mouse shows a phenotype that still has to be categorized as very mild. 

 In summary, the results obtained with knockout animals seem to indicate that ghrelin is not a critical orexigenic factor. Nevertheless, the ghrelin/GHSR pathway plays a role in glucose homeostasis by regulating insulin sensitivity and glucose sensing. If it was confirmed that ghrelin ablation restores the first-phase of insulin secretion, as observed in ghrelin knockout ob/ob mice, [36] this could have clinical relevance, because in humans the loss of first-phase insulin secretion is predictive for the development of type 2 diabetes [111]; therefore, in subjects at risk for type 2 diabetes, treatment with a ghrelin antagonist may prove beneficial. Kelley and colleagues proposed a central pathophysiological construct to describe the altered metabolism associated with insulin-resistant and glucose-intolerant states: the concept of “metabolic inflexibility” [112]. Metabolically normal people can adapt to the discontinuities in fuel availability and utilization present in daily life, whereas diabetic people cannot. Metabolic inflexibility means that insulin-resistant individuals are unable to efficiently increase carbohydrate utilization, even when carbohydrates are plentiful. This is a rewording of the essence of impaired glucose tolerance (and insulin resistance). The results obtained in RQ of GHSR KO mice seem to indicate greater metabolic flexibility and hence improve glucose tolerance. When null mice were fed with either SCD or HFD, their body weights were not different from that of their WT littermates on the same diet. However ghrelin and GHSR null mice were resistant to DIO when were fed with HFD immediately after weaning. But ablation of the ghrelin/GHSR signal does not prevent DIO raised on SCD and then fed with HFD as adults. Considering these data it would be possible to conclude that the loss of ghrelin signalling protects against several fatty diet-induced features of metabolic syndrome and improves insulin sensitivity. But all these results should be taken with caution, considering that the age of exposition and the type of diet seem to be key factors to observe the effect of ghrelin on glucose and insulin metabolism.

### 3.3. Glucose and Insulin Levels in GHSR and Ghrelin Transgenic Animals

There are some studies realized with ghrelin transgenic mouse with overexpression of ghrelin in different tissues or cellular types (Table 6). Many of them presented plasma UAG levels higher than those of their nontransgenic littermates whereas that plasma acylated ghrelin levels did not change [27, 38, 39]. Hence these models can serve to study the role of desacyl as well as acylated ghrelin in the regulation of glucose metabolism and insulin release.

 Iwakura and colleagues developed and analyzed rat insulin II promoter-ghrelin transgenic mice (RIP-G Tg) in which pancreatic ghrelin concentration was higher than that of nontransgenic littermates; moreover in control mice ghrelin was not detected in *β*-cells by immunohistochemistry. Ghrelin transgene driven by RIP was considered to be expressed in *β*-cells, although higher expression levels of ghrelin mRNA were also found in the brain of RIP-G Tg compared with that of nontransgenic littermates. When these animals were subjected to IP-GTT, plasma insulin levels were significantly lower in Tg mice than those in nontransgenic littermates, although there was no significant difference in plasma insulin levels between RIP-G Tg and nontransgenic littermates on the fasting state [39]. The glucose-stimulated insulin secretion of RIP-G Tg was decreased without changes in glucose levels, but there were no abnormalities with the arginine-induced insulin secretion, pancreatic histology, pancreatic insulin mRNA levels, and insulin content in the RIP-G Tg. When the authors did several tests from isolated islets of RIP-G Tg, they found that insulin secretion as well as immunoreactivity of glucose transporter in the pancreatic *β* cell, in RIP-G Tg *β* cells, was indistinguishable from that of nontransgenic littermates, indicating that insulin secretion was not affected by overexpression of ghrelin transgene in vitro, although it was affected in vivo [39]. When these animals were subjected to ITT, they showed a tendency to lower blood glucose levels. Considering the results, the authors suggested that the suppression of insulin secretion of RIP-G Tg is likely due to the effect of desacyl ghrelin on insulin sensitivity [39]. Nevertheless these results do not agree with others studies. 

 Reed et al. created mice with ghrelin overexpressed in neurons using the neuron-specific enolase (NSE) promoter sequences and mouse ghrelin cDNA (NSE-ghrelin). Ghrelin expression in NSE-ghrelin brain tissues was increased compared with wild-type mice; it was also increased to a much smaller extent in liver of these mice, but in stomach or duodenum did not differ from wild-type mice. They worked with two lines of NSE-ghrelin mice: one line with increased circulating AG and UAG (L43) and one line with only UAG (L73). In both lines young NSE-ghrelin mice had normal glucose tolerance; however, L43 NSE-ghrelin mice, but not L73 mice, developed glucose intolerance at 32 week of age. Despite the impaired glucose tolerance in L43 mice, insulin levels did not differ from those of wild-type mice [114]. However, unlike the studies from Iwakura et al. plasma insulin levels did not change after IP-GTT in those animals with high levels of UAG (L73). The differences between both studies can be the consequence of several factors like age or others. In another line of transgenic mice, Zhang et al. created animals in which the ghrelin gene is overexpressed in adipose tissue via the fatty acid-binding protein-4 (FABP4) promoter. Transgenic mice overexpressing the ghrelin gene in adipose tissue demonstrated significant increases in plasma concentrations of UAG, whereas ghrelin remained unchanged. Overexpression of ghrelin from the FABP4 promoter reduced the weight of white adipose tissues and resistance to HFD-induced obesity [26]. Alterations in glucose tolerance and insulin sensitivity tests were detected in FABP4-ghrelin transgenic mice. When these animals were subjected to IP-GTT, glucose levels were significantly lower than in controls; however FABP4-ghrelin transgenic mice had a greater hypoglycemic response to insulin administration than control animals. It seems that UAG improves glucose tolerance and insulin sensitivity, providing more evidences that UAGs play a role in the regulation of glucose metabolism. These data are strengthened by the observation that plasma insulin levels are elevated in transgenic mice [26]. 

 Recently, Bewick et al. generated a mouse model with increased ghrelin expression and production in stomach and brain. Ghrelin transgenic mice exhibited increased circulating AG and total ghrelin which was associated with hyperphagia and increased energy expenditure. These animals were subjected to IP-GTT and ITT; the animals were glucose intolerant due to an inhibition of glucose-stimulated insulin release but without change in insulin sensitivity [113].

## 4. Mechanism of Action

In order to understand how ghrelin can modify glucose and insulin homeostasis, it is important to study the mechanism of action exerted by ghrelin in tissues implied in carbohydrate metabolism. 

### 4.1. Liver

*De novo* synthesis of glucose in the liver from precursors such as lactate, gluconeogenic amino acids, and glycerol is a central mechanism to provide the organism with glucose in times of starvation [115], a natural situation in which ghrelin levels are increased [10, 30]. On the other hand, when glucose is directly available from external resources, gluconeogenesis is dispensable and consequently needs to be shut off. Integration of these events is complex and occurs through various hormonal and nutritional factors. The principal parameters affecting hepatic glucose output are the concentrations of the available glucogenic substrates and the activity of a few regulatory enzymes. The activity of the key gluconeogenic enzymes phosphoenolpyruvate carboxykinase (PEPCK) and G6Pase is regulated by transcriptional and nontranscriptional mechanisms, whereas the third key enzyme fructose-1,6-bisphosphatase (FBPase) is also regulated through competitive inhibition by fructose 2,6-bisphosphate. Insulin is the most important hormone that inhibits gluconeogenesis, through the activation of the insulin receptor (IR). It acts predominantly by suppressing the expression of the genes for the key gluconeogenic enzymes PEPCK and G6Pase [116]. In the H4-II-E-cells (rat hepatoma cell line) and HepG2 cells (human hepatocellular carcinoma cell line) ghrelin was shown to stimulate insulin receptor substrate 1*º*(IRS1) and its downstream molecules, including growth factor receptor-bound protein 2 (Grb2) and mitogen-activated protein kinase (MAPK). Whereas on the other hand, it diminished phospho protein kinase B (pAKT) and phospho-glycogen synthase kinase (pGSK) levels in both cell lines and upregulated gluconeogenesis in H4-II-E-cells by attenuating the effect of insulin on the expression on PEPCK [73]. 

 AKT is a key protein kinase downstream of the insulin receptor [117] and its activation plays a key role in suppressing hepatic gluconeogenesis [118, 119], since GSK-3, which phosphorylate glycogen synthetase (GS) is inhibiting, is phosphorylated by AKT, and this phosphorilation inactivates GSK-3 kinase activity, suppressing hepatic gluconeogenesis resulting in enhanced glycogen deposition [118]. 

 Forkhead box O1 (FOXO1) and peroxisome proliferator activated receptor-*γ*-coactivator (PGC)-1*α* are two transcriptional components that are targets of insulin signalling and that can activate the process of gluconeogenesis in liver. FOXO1 has been shown to bind directly to the promoters of gluconeogenic genes and activate the process of glucose production [120–122]. It is directly phosphorylated by AKT. This phosphorylation results in exclusion of FOXO1 from the nucleus. A second transcriptional component controlled by insulin and having a role in gluconeogenesis is the coactivator PGC-1*α*. It is induced in the liver during fasting and is elevated in several models of diabetes or deficiency in insulin signalling. Notably, expression of PGC-1*α* at physiological levels turns on the entire program of gluconeogenesis [123]. PGC-1*α* hepatic transcription has been reported to be downregulated by AKT activation through forkhead transcription factor FOXO1 phosphorylation and nuclear exclusion [124]. 

 Barazzoni et al. observed that in rats sustained ghrelin administration reduced hepatic phospho/total-AKT (P/T-AKT) and P/T-GSK [86]. These changes in AKT-GSK signalling were associated with enhanced PGC-1*α* expression. Reduced liver AKT signaling could potentially contribute to concomitant blood glucose increments, preserving hepatic glucose production in calorie-restricted status [86]. 

**Figure 1:**
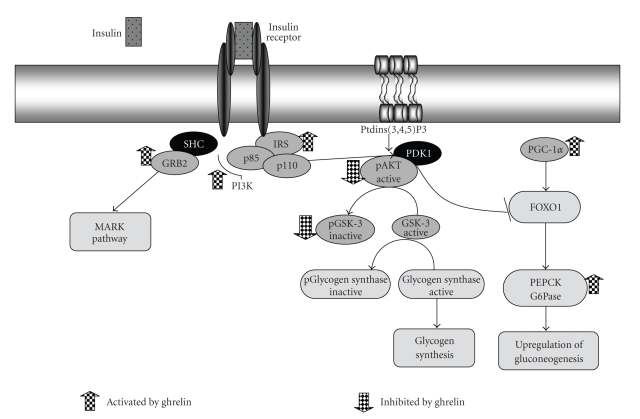
Regulation of hepatic gluconeogenesis and glycogen synthesis by ghrelin. Insulin activates the insulin receptor tyrosine kinase (IR), which phosphorylates and recruits different substrate adaptors. AKT is a key protein kinase downstream of the insulin receptor and its activation plays a key role in suppressing hepatic gluconeogenesis, since GSK-3, which phosphorylate glycogen synthetase (GS) is inhibiting, is phosphorylated by AKT suppressing hepatic gluconeogenesis, resulting in enhanced glycogen deposition. Sustained ghrelin administration in rats reduced hepatic AKT-GSK activation and enhanced PGC-1a expression, suggesting upregulation of gluconeogenesis and downregulation of glyconeogenesis.

The routes that have been modified after treatments with ghrelin in liver and which could modify plasma glucose levels are represented in Figure 1.

### 4.2. Pancreas

Insulin secretion is accurately linked to blood glucose levels in the physiological range. The role of the *β*-cells is to sense an increase in the concentration of nutrients in the blood and to synthesize, package, and release insulin to control blood glucose homeostasis. Various agents as amino acids (particularly arginine and leucine) and fatty acids can increase the secretion of insulin, but only in the presence of facilitating concentrations of glucose (above 3 mM), whilst nonmetabolizable analogues of glucose such as galactose or fructose are inactive as secretagogues [125]. The above fuel secretagogues are initiators of secretion, but there are also other agents including neurotransmitters, glucagon-like peptide (GLP-1), gastric inhibitory peptide (GIP), and pituitary adenylate cyclase-activating polypeptide (PACAP) that act as “potentiators”, enhancing secretion only at permissive concentrations of fuel secretagogues. These molecules usually act via G-protein coupled receptors and the generation of classical second messengers such as cAMP and Ca^2+^ [126]. The first studies about stimulus-secretion coupling in *β*-cells early concluded that glucose must be metabolized by *β*-cells to induce insulin secretion, Ca^2+^ has an essential role in insulin secretion, and pancreatic *β*-cells are electrically excitable [125]. 

**Figure 2:**
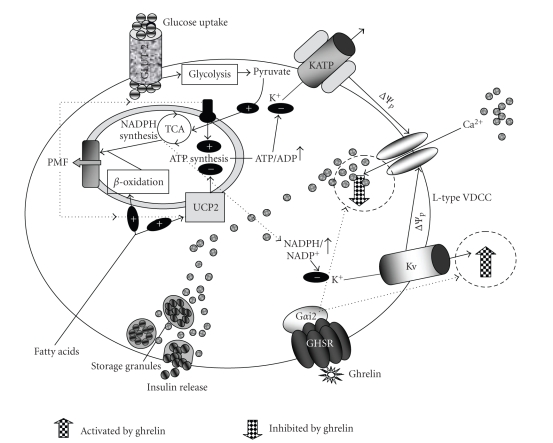
*β*-cell mechanisms of insulin release and its regulation by ghrelin. When the plasma glucose concentration rises, *β*-cells oxidize it. Glucose oxidation establishes a protonmotive force (PMF) that drives ATP synthesis, increasing the ATP/ADP ratio. This causes closure of KATP-channels, depolarisation of the plasma membrane potential (Δ*ψ*p) and Ca^2+^ flux into the cell, triggering insulin release. UCP2 activity dissipates the protonmotive force, lowering ATP/ADP. Ghrelin directly acts on the *β*-cell and via PTX–sensitive mechanisms attenuates glucose-induced [Ca^2+^]i signalling partly through enhancement of TEA-sensitive delayed outward K^+^ currents resulting in decrease plasma insulin levels. PTX catalyzes the ADP-ribosylation of the *α* subunits of the heterotrimeric G proteins Gi, Go, and Gt. This prevents the G proteins from interacting with G protein-coupled receptors on the cell membrane thus interfering with intracellular communication. Since the G*α* subunits remain in their GDP-bound, inactive state, they are unable to inhibit adenylyl cyclase, thus keeping levels of adenylyl cyclase and cAMP elevated. PTX inhibited a number of insulin-stimulated cellular events, such as glucose transport and its metabolism. Antisense oligonucleotide specific for G*α*i2-subunit of G proteins blocks the effects of ghrelin on [Ca^2+^]i and insulin release. Hence ghrelin presumably suppresses glucose-induced insulin release via G*α*i2- and Kv channel–mediated attenuation of Ca^2+^ signalling in *β*-cells.

 Islet *β*-cells are equipped with high-capacity glucose transporters located at the plasma membrane that are known as glucose transporters-2 (GLUT-2) [127]. GLUT-2 is required for efficient glucose-stimulated insulin secretion, as demonstrated by studies in transgenic mice [128, 129]. *β*-cells contain a high Km glucokinase (glucose-phosphorylating hexokinase, GK), which displays strongly cooperative kinetics and has thus been termed the *β*-cell “glucose sensor.” The reduction in *β*-cell GK levels was associated with reduced capacity to secrete insulin in response to glucose [130]. Glucose stimulation of insulin secretion involves two pathways: the triggering of ATP-sensitive K^+^channel- (KATP-) dependent pathway, and the amplifying of KATP channels-independent pathway. The rise in blood glucose induces an increase in *β*-cell glucose metabolism, resulting in increased production of ATP from several sources: glycolysis, mitochondrial glucose oxidation, and active shuttling of reducing equivalents from the cytosol to the mitochondrial electron transport chain. The resultant increase in ATP/ADP ratio inhibits KATP channels, depolarizing the plasma membrane, leading to opening of the voltage-dependent calcium channels (VDCCs), which allows calcium influx. The resultant intracellular calcium concentration ([Ca^2+^]i) rise triggers exocytosis of the insulin-containing granules (reviewed in [131]). However, an expanding bulk of data also makes it apparent that this KATP-channel dependent mechanism of glucose-stimulated insulin secretion does not fully describe the islet glucose response, and signals other than changes in ATP: ADP ratio have been increasingly implicated as important regulators of insulin secretion in recent years. The voltage-dependent K^+^ channels (Kv) [132] are thought to repolarize glucose-stimulated action potentials and inhibit Ca^2+^ entry through voltage-gated Ca^2+^ channels; therefore, Kv channels serve as negative regulators of insulin secretion, and Kv channel antagonists are insulinotropic in a glucosedependent manner. Kv channels are comprised of the pore-forming *α* subunits (Kv2.1 is thought to be the predominant isoform in islet *β*-cells) and regulatory *β*-subunits, analogous to the pore-forming and regulatory subunits of the KATP channel complex. Kv channel *β*–subunits are proposed to act as intracellular redox sensors, and an increase in cytosolic NADPH : NADP ratio in patch-clamped-cells was shown to be associated with an increased rate of inactivation of the Kv channel [133]. Inhibition of Kv channels by NADPH, derived from pyruvate cycling, could serve as a logical complementary mechanism to ATP regulation of KATP channel activity, since suppression of Kv channels would slow membrane repolarization, allowing the effects of KATP channel inhibition to be sustained through a second phase of insulin secretion (Figure 2). But this model is not fully established (reviewed in [134]).

In rat isolated islets, several works showed that endogenous and exogenous ghrelin suppressed glucose-induced insulin release [58, 91, 92, 135]. Dezaki et al. presented ghrelin signalling in *β*-cells. They observed that in rats ghrelin of both endogenous and exogenous origin resulted in pertussis toxin- (PTX-) sensitive decrease in plasma insulin concentrations, contrasting with PTX-insensitive increase in GH levels by ghrelin [58, 136]. PTX catalyzes the ADP-ribosylation of the *α* subunits of the heterotrimeric G proteins G_i_, G_o_, and G_t_. This prevents the G proteins from interacting with G protein-coupled receptors on the cell membrane, thus interfering with intracellular communication. Since the G*α* subunits remain in their GDP-bound, inactive state, they are unable to inhibit adenylyl cyclase, thus keeping levels of adenylyl cyclase and cAMP elevated [137]. In intact cells, PTX inhibited a number of insulin-stimulated cellular events, such as glucose transport and its metabolism. The function of endogenous ghrelin was assessed by the effects of GHSR antagonist in vivo and in rats treated with ghrelin and PTX. In addition, studies with isolated islets from ghrelin-KO mice observed that modified GHRP-6 increased plasma insulin concentrations after IP administration, indicating suppression of insulin levels by endogenous ghrelin. The insulinostatic effect of ghrelin was unaltered by pretreatment with phospholipase C (PLC) inhibitor. However the effects of endogenous and exogenous ghrelin on insulin levels were not observed in PTX-treated rats. In islets isolated from ghrelin-KO mice, glucose-induced insulin release was greater than those from wild-type mice. This enhancement was blunted by pretreatment with PTX. They observed that ghrelin increased Kv currents and that tetraethylammonium (TEA), a Kv channel blocker, eliminated the ability of ghrelin to suppress insulin release. Furthermore, ghrelin treatment-inhibited glucose induced [Ca^2+^]i increases in *β*-cells. All the effects of endogenous and exogenous ghrelin on Kv and [Ca^2+^]i as well as insulin release were blunted in the presence of PTX. This finding suggests that glucose-induced insulin release in islets is markedly decreased by endogenous ghrelin. Endogenous ghrelin in islets restrict glucose-induced insulin release via the following mechanism: ghrelin directly acts on the *β*-cell GH secretagogue receptor and via PTX–sensitive mechanisms attenuates glucose-induced [Ca^2+^]i signalling, partly through enhancement of TEA-sensitive delayed outward K^+^currents [58, 136]. When the islet *β*-cells were treated with the antisense oligonucleotide specific for G*α*_i2_-subunit of G proteins, the effects of ghrelin on [Ca^2+^]i and insulin release were abolished (Figure 2). These findings demonstrate that ghrelin suppresses glucose-induced insulin release via G*α*_i2_- and Kv channel–mediated attenuation of Ca^2+^ signalling in *β*-cells [136]. 

 All these data reveal that endogenous ghrelin in islets acts on *β*-cells to restrict glucose-induced insulin release, at least partly via attenuation of Ca^2+^ signaling, and that this insulinostatic action may be implicated in the upward control of blood glucose. These unique signaling mechanisms and molecules mediating the insulinostatic action of ghrelin on *β*-cells provide potential therapeutic targets for the prevention and treatment of type 2 diabetes and hyperinsulinemia [58, 136].

### 4.3. Adipocytes

**Figure 3:**
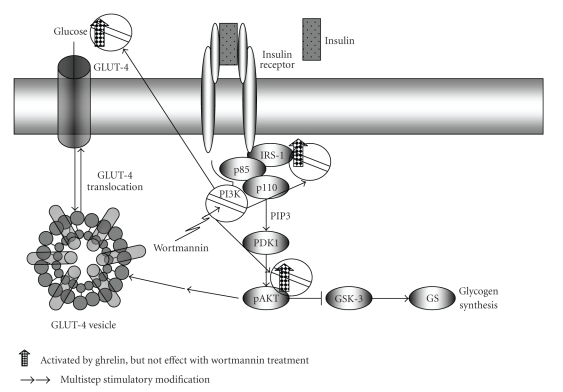
Glucose uptake in adipose tissue and its regulation by ghrelin. Insulin activates the IR, which phosphorylates and recruits different substrate adaptors such as the IRS family of proteins. Tyrosine phosphorylated IRS then displays binding sites for numerous signaling partners. Among them, PI3K has a major role in insulin function, mainly via the activation of the AKT/PKB and the PKCz cascades. Activated AKT induces glycogen synthesis, through inhibition of GSK-3. Insulin stimulates glucose uptake in muscle and adipocytes via translocation of GLUT-4 vesicles to the plasma membrane. GLUT-4 translocation involves the PI3K/AKT pathway. Ghrelin treatment induced increases IRS-1 and AKT phosphorylation, but when the adipocytes were treated with wortmannin, a PI3K inhibitor, completely blocked this ghrelin induced increase in glucose transport and phospho-AKT expression, suggesting that PI3K/AKT activation may mediate the effect of ghrelin on glucose transport in these adipocytes.

The insulin stimulation of glucose uptake in adipose and muscle tissue occurs through a complex and as yet incompletely defined signalling pathway acting through the insulin receptor tyrosine kinase. The primary effect is to promote the movement of the GLUT-4 protein from intracellular storage sites to the plasma membrane. In the basal state, GLUT-4 is localized to a morphologically defined “tubulovesicular system” present in the intracellular compartment, while in the presence of insulin, GLUT-4 is immunolocalized to the plasma membrane of fat cells [138]. The rate-limiting step at which insulin stimulates uptake of glucose in fat is the translocation of GLUT-4 to the plasma membrane [139]. At least two discrete signalling pathways have been implicated in insulin-regulated GLUT-4 translocation. The first involves the lipid kinase phosphatidylinositol 3-kinase (PI3K) [140, 141], and the second involves the proto-oncoprotein c-Cbl [142, 143]. When insulin binds to its receptor induces a conformational change in the receptor and leads to activation of its tyrosine-kinase domain. On activation, the receptor phosphorylates several proximal substrates, including members of the IRS and c-Cbl. Tyrosine-phosphorylated IRS proteins, which are thought to be held in close proximity to the plasma membrane through association with the underlying cytoskeleton, recruit more effectors molecules, such as PI3K, to this location. Two important targets of PI3K in muscle and fat cells that have been shown to have a role in insulin-stimulated GLUT-4 translocation are the AKT and the protein kinase C (PKC). PI3K activates AKT by generating polyphosphoinositides in the inner leaflet of the plasma membrane. This acts as an anchorage site for AKT through its pleckstrin homology domain, thereby bringing it in close proximity to its upstream regulatory kinase, phosphatidylinositol-dependent kinase-1 (PDK-1). The second putative signalling pathway that has been shown to have a role in insulin-stimulated GLUT-4 translocation operates independently of PI3K and involves a dimeric complex that comprises c-Cbl and the c-Cbl-associated protein CAP. Intriguingly, whereas many growth factors trigger the activation of PI3K, AKT, and PKC in many cell types, aspects of the c-Cbl–CAP pathway, including the tyrosine phosphorylation and the expression of CAP, seem to be unique to muscle and fat cells [144]. Patel et al. examined the expression of GHSR1a in discrete adipose tissue depots and while GHSR1a expression was detected in the epididymal and pericardial deposits, it was not found in the perirenal, subcutaneous, and omental deposits. Ghrelin and des-acyl ghrelin did not affect basal deoxyglucose uptake in adipocytes from the epididymal fat deposits. However, treating isolated epididymal adipocytes with ghrelin in the presence of insulin increased insulin-stimulated deoxyglucose uptake. Des-acyl ghrelin had no significant effect on insulin-stimulated deoxyglucose uptake in isolated epididymal adipocytes. Ghrelin had no effect on basal deoxyglucose uptake in isolated perirenal adipocytes, which do not express the GHSR1a mRNA. As expected, insulin increased glucose uptake, but ghrelin in the presence of insulin did not further increase this response. Furthermore, des-acyl ghrelin did not increase insulin-stimulated deoxyglucose uptake in perirenal adipocytes. These data suggest that ghrelin may act synergistically to potentiate insulin-stimulated glucose uptake and may improve insulin sensitivity [145]. Interestingly, ghrelin did not affect insulin-stimulated glucose uptake in perirenal adipocytes, which do not express GHSR1a, and des-acyl ghrelin, which does not bind to GHSR1a, did not influence insulin-stimulated glucose uptake in epididymal adipocytes The effects of ghrelin on adipocyte glucose uptake might be expected to result in fatty acid accumulation and an increase in adiposity in the long term [145]. Kim and colleagues incubated terminally differentiated 3T3-L1 adipocytes with insulin or/and ghrelin overnight and assayed glucose transport. Insulin and ghrelin increased glucose transport and the cotreatment of insulin and ghrelin induced a further increase in glucose transport. In addition, ghrelin treatment induced increases IRS-1 and AKT phosphorylation, but when the adipocytes were treated with wortmannin, a PI3K inhibitor, completely blocked this ghrelin induced increase in glucose transport and phospho-AKT expression [146], suggesting that PI3K/AKT activation may mediate the effect of ghrelin on glucose transport in these adipocytes (Figure 3). 

All these data suggest that the direct effects of ghrelin on insulin-stimulated glucose uptake are mediated by the GHSR1a and PI3K/AKT activation.

## 5. Pharmacological Uses of Ghrelin on Glucose-Insulin Homeostasis

Overt diabetes mellitus is defined clinically by fasting or postprandial hyperglycemia or an abnormally increased glucose excursion in response to a defined glucose load. Insulin resistance, measured as impaired glucose disposal in a hyperinsulinemic-euglycemic clamp study, is one of the earliest detectable disorder and is considered a cardinal pathophysiologic feature [147]. Fasting hyperinsulinemia is also present early in the disease process and is thought to be a compensatory mechanism to maintain euglycemia in the setting of insulin resistance [148]. Even while maintaining a healthy lifestyle, most patients need pharmacological intervention which might consist of one or a combination of the following oral medications: sulfonylureas, glinides, incretin mimetics, *α*-glucosidase inhibitors, metformin, or thiazolidinediones. However 30%–40% of patients are not adequately controlled by these therapies and require subcutaneous insulin injections intended to restore normoglycemia, but they can inadvertently lead to hypoglycemia, a potentially fatal consequence. Thus, new drugs and novel methods of treatment are needed. Among diabetic patients, 10%–20% fall into the category of insulin-dependent diabetes mellitus (IDDM) or type 1 diabetes, which generally appears before age 40, frequently in adolescence, and results from autoimmune destruction of insulin producing pancreatic *β*-cells. Type 1 diabetic patients depend on insulin administration for their survival. Noninsulin-dependent diabetes mellitus (NIDDM) or type 2 diabetes is far more common than IDDM, affecting 80%–90% of diabetic patients. The prevalence of obesity and type 2 diabetes continues to increase at alarming rates [149]. Type 2 diabetes is a prototypic complex, polygenic disease with a strong heritable component, which is also heavily influenced by environmental factors, especially diet and physical activity. It appears that altered communication among tissues and loss of the ability of tissues to adapt to changing metabolic states play a critical role in the altered glucose homeostasis that leads to the development of type 2 diabetes. It is characterized by a combination of factors that affect the organism's ability to respond to insulin. The condition has two hallmark features: (1) insulin resistance and (2) compromised function of the pancreatic *β*-cell, such that insulin secretion is insufficient to counterpart the degree of insulin resistance. There is general agreement that type 2 diabetes, unlike IDDM, is tightly associated with obesity. Over 80% of individuals with type 2 diabetes are obese. However, only 10% of obese individuals are diabetic. In the prediabetic phase, when insulin resistance has already begun, the *β*-cell actually hypersecretes insulin despite normal blood glucose levels. What has defied explanation is precisely what causes this insulin resistance in the first place and how it relates in a temporal sense to the accompanying hyperinsulinemia. 

Ghrelin receptor modulation could be clinically useful for different situations related with glucose-insulin homeostasis (Table 7). Several works demonstrated that ghrelin concentrations are negatively associated with fasting insulin levels, the prevalence of type 2 diabetes and insulin resistance in humans, regardless of race [102, 150, 151]. The data obtained since ghrelin discovery show that both the acylated and unacylated molecules are actively involved in the acute and long-term control of glucose metabolism and insulin sensitivity in humans, which might enable new treatment modalities for the many disorders in which insulin sensitivity is disturbed. Thus, pharmacological, immunological, and genetic blockade of ghrelin or ghrelin action in pancreatic islets all markedly enhanced glucose-induced insulin release and improve the diabetic condition. Hence ghrelin inhibition could be useful for the treatment of diabetes [152, 153]. 

 On the other hand the ability to efficiently build fat reserves in times of nutritional abundance appears to have resulted from evolutionary pressure to protect against subsequent periods of food scarcity. The tendency to efficiently store fat in times of caloric excess appears to have become paradoxically maladaptive in settings of continuous food availability, as indicated by the present epidemic of obesity in Western societies. The data obtained in the last years seem to indicate that ghrelin may be one of the primary mechanisms by which an individual can sense changes in nutrient availability and trigger biological responses that modulate the efficiency of energy storage (and particularly fat deposition) during periods of fuel overflow or after a period of scarcity of nutrients. At present, ghrelin is the only peripheral orexigenic factor that is effective upon its intravenous administration [81]. Put in this context, the blockade of the route of ghrelin could prove useful in controlling adiposity in human obesity, as blockers of the orexigenic signal from the gastrointestinal tract to the brain, or diminishing the ability to efficiently store fat reserves. Inverse agonists of the ghrelin receptor, by blocking the constitutive receptor activity, might lower the set-point for hunger between meals [110, 154]. All these data suggest that ghrelin-ghrelin receptor modulation has the potential to improve the diabetic condition by promoting glucose-dependent insulin secretion and promoting weight loss. 

 In contrast, ghrelin may be useful as an orexigenic agent for the treatment of eating disorders such as anorexia nervosa. Administration of ghrelin can stimulate appetite and improve the nutritional status of these patients. However, plasma ghrelin concentrations in anorexia nervosa are high, indicating a situation of ghrelin resistance [100]. In fact, circulating ghrelin levels have been found altered in different clinical situations, like renal failure or hepatic failure [101, 155]. Ghrelin-derived drugs could also be useful in all the clinical situations associated with cachexia, such as malignancy, advanced cardiac failure, renal failure, postoperative patients, and human immunodeficiency virus-lipodystrophy. In Table 8 we summarize putative ghrelin effects on glucose-insulin homeostasis and related physiological actions. 

 In summary, there are multiple studies suggesting that ghrelin could have an important function in glucose homeostasis and insulin release and probably insulin action. At least theoretically ghrelin and/or its signalling manipulation could be used for the treatment or prevention of diseases of glucose homeostasis such as type 2 diabetes.

## Figures and Tables

**Table 1:** Acute effects of ghrelin administration on glucose-insulin homeostasis in different species and metabolic situations. IV: intravenous; O: oral.

Species	Treatment	Dose	Food before experiment	Treatment duration	Plasma glucose or GIR	Plasma insulin	Reference
Health humans	1 IV AG injection versus 1 IV placebo injection	1 *μ*g AG/kg	Fasting overnight	3 hours	Enhanced	Decreased	[65]

Health humans	1 IV AG injection versus 1 IV placebo injection	3.3 *μ*g AG/kg	Fasting overnight	3 hours	Enhanced	Decreased	[68]

Health humans	1 IV AG injection versus 1 IV placebo injection	1 *μ*g AG/kg	Fasting overnight	2 hours	Enhanced	Decreased	[67, 81]

Health humans	1 IV AG injection + O-GTT versus O-GTT	1 *μ*g AG/kg + 100 g glucose	Fasting overnight	2 hours	Not change	Not change	[81]
	1 IV AG injection + FFA versus FFA	1 *μ*g AG/kg + 10% FFA	Fasting overnight	2 hours	Not change	Decreased	
	1 IV AG injection + arginine versus arginine	1 *μ*g AG/kg + 0.5 g arginine/kg	Fasting overnight	2 hours	Enhanced	Decreased	

Health humans	1 IV AG injection versus 1 IV placebo injection	1 *μ*g AG/kg	Fasting overnight	2 hours	Enhanced	Decreased	[67]
	1 IV UAG injection versus 1 IV placebo injection	1 *μ*g UAG/kg	Fasting overnight	2 hours	Not change	Not change	
	1 IV AG injection + UAG versus 1 IV placebo injection	1 *μ*g AG/kg + 1 *μ*g UAG/kg	Fasting overnight	2 hours	Not change	Not change	

Health humans	IV AG infusion versus IV placebo infusion	5 pmol AG/kg/min	Fasting overnight	3 hours	Enhanced	Enhanced	[77]

Health humans	IV AG infusion versus IV placebo infusion Both with pancreatic clamp + hyperinsulinemic-euglicemic clamp + glucose adjustable	5 pmol AG/kg/min	Fasting overnight	5 hours	During clamp GIR diminished with ghrelin	Not change	[71]

Hypopituitary humans	1 IV AG or UAG injection versus 1 IV placebo injection	1 *μ*g AG or UAG/kg	Fasting overnight	2 hours	Enhanced	Not change	[82]
	1 IV AG + UAG injection versus 1 IV placebo injection	1 *μ*g AG/kg + 1 *μ*g UAG/kg	Fasting overnight	2 hours	Not change	Diminished	

Hypopituitary humans	IV AG infusion versus IV placebo infusion Both with hyperinsulinemic-euglicemic clamp	5 pmol AG/kg/min	Fasting overnight	5 hours	Basal period enhanced, during clamp GIR diminished	Not change	[78]

Gastrectomized humans	IV AG infusion versus IV placebo infusion Both with hyperinsulinemic-euglycemic clamp	5 pmol AG/kg/min	Fasting overnight	5 hours	Diminished GIR	Not change	[80]

	IV AG infusion versus IV placebo infusion Both with IV-GTT infusion	1 ng AG/kg/h + 13.3 mg glucose/kg/min	24-hour fasting	40 minutes	Not change	Not change	
Normal rats	IP AG infusion versus IV placebo infusion Both with IV-GTT infusion	1 ng AG/kg/h + 13.3 mg glucose/kg/min	24-hour fasting	40 minutes	Enhanced	Diminished	[70]
	IV AG infusion versus IP placebo infusion Both with IP-GTT infusion	1 ng AG/kg/h + 13.3 mg glucose/kg/min	24-hour fasting	40 minutes	Not change	Not change	
	IP AG infusion versus IP placebo infusion Both with IP-GTT infusion	1 ng AG/kg/h + 13.3 mg glucose/kg/min	24-hour fasting	40 minutes	Enhanced	Diminished	

Normal rats	1 IV UAG injection + IV-GTT versus IV-GTT	30 nmol UAG/kg + 1 g glucose/kg	Fasting overnight	50 minutes	Not change	Enhanced	[83]
	1 IV AG injection + IV-GTT versus IV-GTT	30 nmol UAG/kg + 1 g glucose/kg	Fasting overnight	50 minutes	Not change	Not change	

Rats with hepatic vagotomy	IP AG infusion versus IP placebo infusion Both with IP-GTT infusion	1 ng AG/kg//h +13.3 mg glucose/kg/min	24-hour fasting	40 minutes	Not change	Not change	[70]

Mice ddY	1 IP AG injection versus 1 IP placebo injection Both with IP-GTT	1 and 10 nmol AG/kg + 1 g glucose/kg	Fasting overnight	2 hours	Enhanced	Decreased	[58]
	1 IP AG injection versus 1 IP placebo injection	1 nmol/kg	Fasting overnight	2 hours	Enhanced		

C57BL/6J mice	1 IV AG injection + IV-GTT versus IV-GTT	50 nmol AG/kg + 1g/kg	3-hour fasting	50 minutes	Not change	Diminished	[84]

GH-deficient little mice	1 IP AG injection versus 1 IP placebo injection	1 nmol AG/kg	Fasting overnight	30 minutes	Enhanced		[58]

Obese humans	1 IV AG injection versus IV placebo injection	1 *μ*g AG/kg	Fasting overnight	2 hours	Not change	Not change	[72]

**Table 2:** Chronic effects of ghrelin administration on glucose-insulin homeostasis in different species.

Species	Treatment administration	Dose	Food during experiment	Duration treatment	Plasma glucose levels	Plasma insulin levels	Reference
Mice ddy	1 IP AG injection/12-h	3 nmoles AG/mouse/injection	*Ad libitum* HFD	5-day	Not change	Enhanced	[75]
Tundra vole	1 IP AG injection/day	10 *μ*g AG/kg/day	*Ad libitum* SCD	4-day	Enhanced		[69]
Sprague-Dawley rats	1 ICV AG injection/12-h	1 *μ*g AG/rat/injection	*Ad libitum* SCD	3-day	Not change	Not change	[88]
Wistar rats	1 ICV ghrelin injection/day	1 *μ*g AG/rat/day	*Ad libitum* SCD	5-day	Not change	Enhanced	[89]
	ICV ghrelin infusion	2.5 nmol AG/rat/day	*Ad libitum* SCD	6-day	Not change	Not change	[87]
	1 SC AG injection/12-h	0.2 ug AG/injection	*Ad libitum* SCD	4-day	Enhanced	Not change	[85]

**Table 3:** Results obtained with cellular cultures and pancreatic perfusion that contribute to data about ghrelin role on glucose and insulin metabolism.

Cellular type/Perfusion	Treatment	Dose	Insulin release	Glucose output	Reference
Islets from normal rats	AG + glucose versus glucose	10^−12^M AG + 2.8 mM glucose	Not change		[54]
	AG + glucose versus glucose	10^−12^M AG + 8.3 mM glucose	Enhanced		

Islets from normal rats	AG + glucose versus glucose	10^−8^M AG + 2.8 mM glucose	Not change		[58]
	AG + glucose versus glucose	10^−8^M AG + 8.3 mM glucose	Diminished		

Islets from normal rats	AG + glucose versus glucose	10 nM AG + 20 mM glucose	Diminished		[92]
	UAG + glucose versus glucose	1 *μ*M + 20 mM glucose	Not change		
	AG + glucose + YIL-781 versus glucose	10 nM AG + 20 mM glucose + 1 *μ*M YIL-781	Not change		
	Glucose + YIL-781 versus glucose	20 mM glucose + 1 *μ*M YIL-781	Not change		

Islets from normal rats	GHRP-6 versus placebo	1 *μ*M GHRP-6	Enhanced		[58]
	SPA versus placebo	1 *μ*M SPA	Enhanced		

Ghrelin KO mouse islets	Glucose ghrelin KO versus glucose wildtype	8.3 mM and 16.7 mM glucose	Enhanced		[91]

Min 6 cells	AG + glucose versus glucose	1–10 nM AG + 22.2 mM glucose	Diminished		[93]

Hepatocytes from pigs	AG versus placebo	100 nM AG		Enhanced	[96]
	UAG versus placebo	100 nM UAG		Diminished	
	UAG + AG versus AG	100 nM AG + 100 nM UAG		Diminished	

Pancreas of rat perfused in situ	Ghrelin + glucose versus glucose	10 nM ghrelin + 5.5 mM glucose	Not change		[90]
	Ghrelin + glucose versus glucose	10 nM ghrelin + 9 mM glucose	Diminished		

Pancreas of rat perfused in vitro	Ghrelin + glucose versus glucose	10 nM ghrelin + 8,3 mM glucose	Diminished		[91]
	GHRP-6 + glucose versus glucose	1 *μ*M GHRP-6 + 8.3 mM glucose	Enhanced		
	UAG + glucose versus glucose	10 nmol/l UAG + 8.3 mM glucose	Not change		

**Table 4:** Effects of GHSR antagonists on glucose and insulin levels.

Species	Treatment Administration	Dose	Feeding	Measurement blood samples	Plasma glucose levels	Plasma insulin levels	Reference
Mice ob/ob	1 IP GHSR antagonist injection/12 hours versus 1 IP placebo injection	200 nmol GHRP-6/mouse	*Ad libitum* SCD	Endpoint 6-day	Diminished	Diminished	[75]

Mice ddY	1 IP GHSR antagonist injection versus 1 IP placebo injection	10 *μ*mol GHRP-6/kg	Fasting overnight	Time course 2 hours	Diminished	Enhanced	
	1 IP GHSR antagonist injection versus 1 IP placebo injection	1 *μ*mol SPA/kg	Fasting overnight	Time course 2 hours	Diminished	Enhanced	[58]
	1 IP GHSR antagonist + ghrelin injection versus 1 IP ghrelin injection	1 *μ*mol GHRP-6/kg +10 nmol ghrelin/kg	Fasting overnight	End point 0.5 hours	Diminished		

Normal rats	1 IP GHSR antagonist injection versus 1 IP placebo injection	10 *μ*mol GHRP-6/kg	Fasting overnight	End point 0.5 hours		Enhanced	[91]
Gastrectomized rats	1 IP GHSR antagonist injection versus 1 IP placebo injection	10 *μ*mol GHRP-6/kg	Fasting overnight	End point 0.5 hours		Enhanced	

Normal rats	Oral GHSR antagonist + IP-GTT versus IP-GTT	10 mg YIL-781/kg +2 g glucose/kg	Fasting overnight	Time course 6 hours	Diminished	Enhanced	[92]
	Oral GHSR antagonist versus placebo	30 mg YIL-781/kg	Fasting overnight	Time course 6 hours	Diminished		
DIO rats	Oral GHSR antagonist +IP-GTT versus placebo + IP-GTT	3 mg YIL-781/kg +2 g glucose/kg	Fasting overnight	Time course 6.5 hours	Diminished		

**Table 5:** Glucose and insulin levels in GHSR-, ghrelin-, and double-knockout animals.

Null mice	Treatment	Dose	Food before/during experiment	Measurement blood samples	Plasma glucose levels	Plasma insulin levels	Reference
Ghrelin	KO versus wildtype		SCD 4–20 weeks old	Endpoint	Not change	Not change	[35]

Ghrelin	KO versus wildtype		SCD 4–10 weeks of age	Endpoint	Not change	Not change	[103]

Ghrelin	IP-GTT, KO versus wildtype	2 g glucose/kg	SCD, fasted	Time course-2 hours	Diminished	Enhanced	[91]
	KO versus wildtype		SCD, fed	Endpoint	Endpoint	Not change	
	KO HDF versus KO SCD		HFD 8–12 weeks old	Endpoint	Not change	Enhanced	
	IP-GTT, KO HFD versus KO SCD	2 g glucose/kg	HFD 8–12 weeks old	Time course-2 hours	Not change	Enhanced	

Ghrelin	IP-GTT, KO versus wildtype	2.5 g glucose/kg	SCD 8-week old	Time course-2 hours	Diminished	Enhanced	[36]

Ghrelin	KO versus wildtype		SCD, 6 hours fast	Endpoint	Not change	Not change	[37]

	AG versus saline	2.5 g glucose/kg + 1 IP injection of 150 nmol AG/kg	SCD 8-week old, 18 h fast	Time course-2 hours	Enhanced	Diminished	[36]
	ITT, KO versus wildtype	0.75 U/kg	SCD 8-week old, 8h fast	Time course-2.5 hours	Diminished		
Ghrelin	Hyperinsulinemic-euglycemic clamp, KO versus wildtype		SCD 8-week old		GIR enhanced during clamp		
	KO versus wildtype		SCD 12-week old	Endpoint	Not change	Not change	
	KO.ob/ob versus wildtype.ob/ob		SCD 12-week old	Endpoint	Diminished	Enhanced	
	KO.ob/ob versus wildtype.ob/ob		SCD 12-week old, 24 hours fast	Endpoint	Diminished	Not change	

Ghrelin	IP-GTT, KO versus wildtype	2 g glucose/kg	SCD, 6 hours fast	Time course-2 hours	Not change	Not change	[37]
	ITT, KO versus wildtype	1 U/kg	SCD, 6 hours fast	Time course-2 hours	Not change	Not change	

Ghrelin	O-GTT, KO DIO versus wildtype DIO	1 g glucose/kg	HFD 8–23 weeks old, 16 hours fast	Time course-2 hours	Not change	Diminished	[33]

Ghrelin	KO versus wildtype		10-week SCD + 40 days on 50% caloric restriction with SCD	Time course every 2 days	2–16 day diminished		[34]

	KO DIO versus wildtype DIO		HFD 8–23 weeks old	Endpoint	diminished	Diminished	
	IPGTT, KO DIO versus wildtype DIO	1 g glucose/kg	HFD 8–23 weeks old, 16 hours fasted	Time course-2 hours	Not change	Diminished	[33]

Ghrelin	Hyperinsulinemic-euglycemic clamp, KO DIO versus wildtype DIO	10 mU insulin/kg + constant infused insulin 5 mU/kg/min + infused 20% glucose	HFD 8–23 weeks old, 16 hours fast	Time course-1.5 hours	GIR enhanced	
	Hyperglycemic clamp KO DIO versus wildtype DIO	20% glucose at rates that stabilized blood glucose at 300 mg/dl	HFD 8–23 weeks old, 16 hours fasted	Time course-1.5 hours		Diminished	
	KO versus wildtype		24-week SCD	Endpoint	Not change	Not change	
	KO versus wildtype		24-week SCD/18 h-fasting	Endpoint	Diminished	Diminished	
GHSR	KO versus wildtype		10-week SCD + 40 days on 50% caloric restriction with SCD	Time course every two days	2–28 day diminished		[34]
	KO versus wildtype		14-week SCD +10-week HF + 18 h-fasting	Endpoint	Not change	Not change	
	KO versus wildtype		14-week SCD +10-week HF	Endpoint	Not change	Not change	

	IP-GTT KO versus wildtype	2 g glucose/kg	SCD, 6 hours-fasting	Time course 2 hours	Not change	Not change	[37]
GHSR	ITT KO versus wildtype	1 U/kg	SCD, 6 hours-fasting	Time course 2 hours	Not change	Not change	
	KO versus wildtype		SCD, 6 hours-fasting	Endpoint	Not change	Not change	

GHSR	KO versus wildtype		SCD 4–19 weeks old	Endpoint	Diminished	Diminished	[104]

GHSR	KO versus wildtype		SCD 8-week old	Endpoint	Diminished	Diminished	[105]

Ghrelin + GHSR	IP-GTT dKO versus wildtype	2 g glucose/kg	SCD, 6 hours-fasting	Time course-2 hours	Not change	Not change	[37]
	dKO versus wildtype		SCD, 6 hours-fasting	Endpoint	Not change	Not change	
	ITT dKO versus wildtype	1 U/kg	SCD, 6 hours-fasting	Time course-2 hours	Not change	Not change	

**Table 6:** Relation between overexpression of ghrelin in different tissues or cellular types and glucose-insulin levels.

Transgenic animals	Ghrelin levelstransgenic versus wildtype	Treatment	Food before/during experiment	Treatment duration	Plasma glucose levels transgenic versus wildtype	Plasma insulin levels transgenic versus wildtype	Reference
Ghrelin is overexpressed inadipose tissue	AG: not change UAG: enhanced	Nothing	*Ad libitum*	Endpoint		Enhanced	[26]
		IP-GTT	16-h fast	2.5 hours	Diminished		
		IP-ITT	16-h fast	2.5 hours	Diminished		

Ghrelin is overexpressed in stomach and hypothalamus	AG: enhanced UAG: enhanced	IP-GTT	18-h fast	2.5 hours	Enhanced	Diminished	[113]
		IP-ITT	4-h fast	2.5 hours	Not change	Not change	

Ghrelin is overexpressed in pancreas	AG: not change UAG: enhanced	Nothing	Overnight fast	Endpoint	Not change	Not change	[39]
		IP-GTT	Overnight fast	2 hours	Not change	Diminished	
		IP-GTT	Overnight fast	2 hours	Not change	Diminished	
		ITT	4-h fast	3 hours	Not change	Not change	

Ghrelin is overexpressed in hypothalamus, cortex and liver	AG: enhanced UAG: enhanced	IP-GTT	20-h fast	2 hours	Enhanced	Not change	[114]
Ghrelin is overexpressed in hypothalamus, cortex and liver	AG: not change UAG: enhanced	IP-GTT	20-h fast	2 hours	Not change	Not change	

Ghrelin is overexpressed in wide variety of tissues	AG: not change UAG: enhanced	Nothing	*Ad libitum*	Endpoint	Not change	Not change	[27]

**Table 7:** Potential therapeutic uses of ghrelin agonists and antagonists on glucose-insulin homeostasis.

Ghrelin agonists	Ghrelin antagonists
Insulinoma	Type 2 Diabetes mellitus
Anorexia nervosa	Metabolic syndrome
Cachexia of malignancy	Obesity

**Table 8:** Summary of putative ghrelin effect on glucose-insulin homeostasis and related physiological actions.

Ghrelin effects on glucose-insulin homeostasis
Increase glycemia
Decrease insulinemia
Increase food intake
Increase body weight and adiposity percentage
Increase GH secretion
